# The role of heritability in mapping expression quantitative trait loci

**DOI:** 10.1186/1753-6561-1-s1-s86

**Published:** 2007-12-18

**Authors:** Song Huang, David Ballard, Hongyu Zhao

**Affiliations:** 1 Program of Computational Biology and Bioinformatics, Yale University, New Haven, Connecticut 06520, USA; 2 Department of Epidemiology and Public Health, Yale University, New Haven, Connecticut 06520, USA; 3 Department of Genetics, Yale University, New Haven, Connecticut 06520, USA

## Abstract

Gene expression, as a heritable complex trait, has recently been used in many genome-wide linkage studies. The estimated overall heritability of each trait may be considered as evidence of a genetic contribution to the total phenotypic variation, which implies the possibility of mapping genome regions responsible for the gene expression variation via linkage analysis. However, heritability has been found to be an inconsistent predictor of significant linkage signals. To investigate this issue in human studies, we performed genome-wide linkage analysis on the 3554 gene expression traits of 194 Centre d'Etude du Polymorphisme Humain individuals provided by Genetic Analysis Workshop 15. Out of the 422 expression traits with significant linkage signals identified (LOD > 5.3), 89 traits have low estimated heritability (*h*^2 ^< 10%), among which 23 traits have an estimated heritability equal to 0. The linkage analysis on individual pedigree shows that the overall LOD scores may result from a few pedigrees with strong linkage signals. Screening gene expressions before linkage analysis using a relatively low heritability (*h*^2 ^< 20%) may result in a loss of significant linkage signals, especially for *trans*-acting expression quantitative trait loci (49%).

## Background

Gene expression has been studied as heritable and complex traits in genetic linkage studies [1]. To dissect such traits, a fundamental question is what proportion of the variation of the gene expression can be attributed to genetic factors. Broad heritability, the proportion of a phenotypic variation explained by genetic factors, can be estimated to address this question, especially for selecting traits of interest before mapping [2]. Evidence for a significant heritable component of expression traits has been found in heritability studies on humans [3,4], yeast [5] and mice [3]. Various aspects of heritability in gene expression were systematically reviewed by Stamatoyannopoulos and about 10% to 50% of transcripts' between-individual variation was found to be heritable differences [6]. Most recently, Petretto et al. studied the influence of heritability on the detection of *cis*- and *trans*-acting expression quantitative trait loci (eQTLs) using the BHX/HXB panel of rat recombinant inbred strains in a tissue specific context [7] and concluded that heritability alone is not a reliable predictor of whether an eQTL will be detected for an expression trait. However, compared to the rat crosses in inbred lines, human samples may have more experimental variability due to cell line handling and more extreme allele frequencies at the loci of interest. Thus, whether a similar conclusion can be drawn for human samples deserves further investigation. Using data for the 14 Centre d'Etude du Polymorphisme Humain (CEPH) Utah families provided by Genetic Analysis Workshop 15 (GAW15), we explored the relationship between the heritability of a gene's expression level and the power to identify regions regulating its expression. We also investigated the contributions from each individual family [8].

## Methods

### Data set

We used 3554 expression profile traits together with marker data on 2819 autosomal single-nucleotide polymorphisms (SNPs) from the 14 CEPH families, consisting of 194 individuals, provided by GAW15. Sex-specific Rutger's genetic maps were provided by Sung et al. [9].

### Heritability estimates and genome-wide linkage analysis

To study the relationship between the heritability of a gene expression and the detection of eQTLs, we estimated narrow-sense trait heritability assuming no dominance effect using the standard variance-component model [10]. Genome-wide linkage analysis was conducted using MERLIN-REGRESS and MERLIN variance-components method without covariate adjustment [10,11]. The estimated heritability of each expression trait, sample mean, and variance from all 14 pedigrees were used as population trait distribution parameter estimators in the linkage analysis with MERLIN-REGRESS. The error-checking algorithm implemented in MERLIN was applied before linkage analysis was conducted.

To study whether heritability can be a reliable predictor for linkage signals, we selected gene expression traits that show linkage signal but with low heritability estimates. We applied permutation procedures to examine whether the observed linkage signals are false signals. To adjust for multiple comparisons, the observed genome-wide maximum LOD score for a gene expression was compared to the genome-wide maximum LOD score for the gene expression from a permuted sample. The *p*-value is the number of times that the genome-wide maximum LOD score from permuted data is greater or equal to the observed genome-wide maximum LOD score out of 1000 permutations. To preserve pedigree structures, permutations were performed within each pedigree.

## Results

### Genome-wide linkage analysis

**Figure 1:**
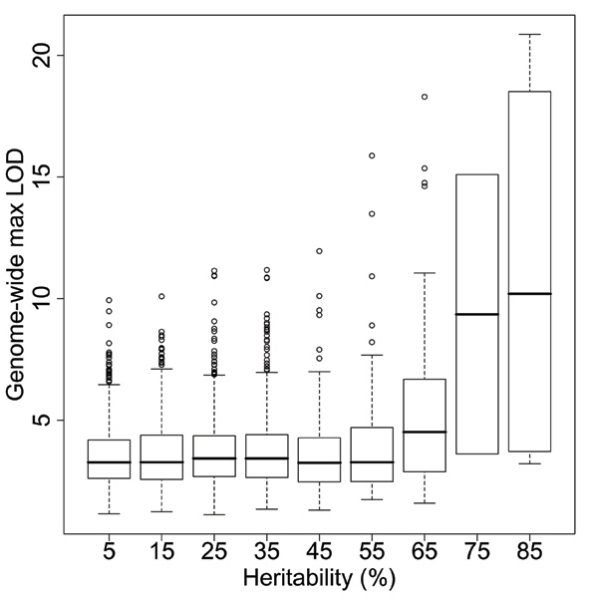
Genome-wide maximum LOD scores vs. heritability estimates.

LOD > 5.3 was used as the significance threshold (corresponding to a point-wise *p*-value < 3.9 × 10^-7^, and a genome-wide *p*-value = 0.001) as in [8]. We identified 422 gene expressions with significant linkage signals, 25 of which have *h*^2 ^> 50%. A positive correlation (0.68) was observed between the heritability estimates and the LOD scores for these 25 gene expressions. However, this correlation dropped to 0.12 when all 3554 gene expressions were considered. Moreover, among the 880 gene expressions that have *h*^2 ^< 10%, 89 have significant linkage signals, including 23 traits with an estimated heritability of 0 (Fig. 1).

### Heritability as predictor for linkage analysis

**Figure 2:**
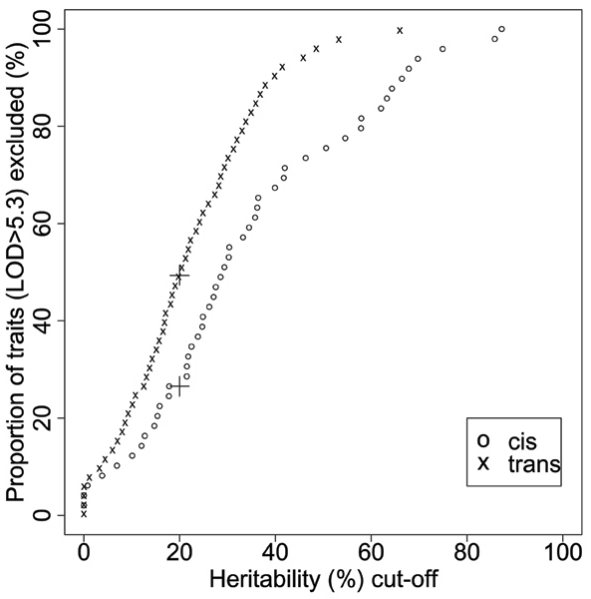
**The proportion of traits with significant eQTL(LOD > 5.3) excluded if filtered by heritability**. The "+" signs highlight the percentage of eQTLs excluded by using *h*^2 ^< 20% as a filter (49% for *trans*- and 27% for *cis*-eQTLs).

We broadly defined the eQTLs that locate on the same chromosome as the mapped gene expressions as *cis*-acting eQTLs, and *trans*-acting eQTLs otherwise. Therein, 422 gene expressions with linkage signals were grouped into a *cis*-acting group of 49 expressions and a *trans*-acting group of 373 expressions. Ten percent of the *cis*-acting gene expressions and 23% of the *trans*-acting gene expressions had an estimated heritability less than 10%. For gene expression in the *cis*-acting group, the estimated heritability has a mean of 0.35, which is higher than the 0.22 mean of the *trans*-acting group. If we screened gene expressions using *h*^2 ^> 20% as the first step before linkage analysis, 183 out of the 373 (49%) gene expressions with *trans*-acting eQTLs and 13 out of the 49 (27%) gene expressions with *cis*-acting eQTLs would be excluded at the screening stage (Fig. 2). Permutation analysis on gene expressions with the bottom five heritability estimates (all with *h*^2 ^= 0) among the 422 gene expression with linkage signals indicated that the observed linkage signals are not false positives at the 0.05 significance level (Table 1).

**Table 1:** Linkage analysis with all 14 pedigrees

Genes^a^	*cis *or *trans*	*h*^2b^	Reg^c^	VC^d^	*p*-value^e^
*OR6A2*	*trans*	0	5.83	0.31	0.027
*NDUFB4*	*trans*	0	7.05	0.59	0.038
*MAP3K6*	*trans*	0	7.39	1.39	0.004
*RGS1*	*trans*	0	7.66	0.85	0.014
*ARPC5L*	*cis*	0	8.91	1.12	0.015
*CHI3L2*	*cis*	0.75	15.10	12.97	0.001
*ITGB1BP1*	*cis*	0.66	11.05	8.35	0.001
*HLA-DPB1*	*cis*	0.70	14.62	8.53	0.001
*HLA-DQB1*	*cis*	0.86	16.16	14.52	0.001
*ZP3*	*cis*	0.87	20.86	14.37	0.001

^a^From the 23 genes with heritability estimates equal to 0, five genes with the highest MERLIN-REGRESS LOD scores are shown in the top five rows; from 422 genes with significant MERLIN-REGRESS LOD scores, five genes with the highest heritability estimates are shown on the bottom five rows as a comparison.

^b ^The trait heritability estimations.

^c ^The genome-wide maximum LOD scores obtained from MERLIN-REGRESS method.

^d ^The genome-wide maximum LOD scores obtained from MERLIN variance component method.

^e^*p*-value from the permutation procedures.

### Between-pedigree and within-pedigree variation

Based on the heritability estimation, we separated out 89 gene expressions with significant linkage signals but that have *h*^2 ^< 10%. We further assessed the contribution of each pedigree to the LOD score of the selected 89 gene expressions through the regression-based linkage analysis, where population trait mean, variance, and heritability estimations obtained from all 14 pedigrees were used. We found that 72 out of the 89 selected gene expressions had at least one pedigree contributing a LOD score greater than one (Table 2). This suggests that some pedigrees contribute to the overall significance and explains why high LOD scores were observed for these gene expressions with low heritability estimates.

**Table 2:** Linkage analysis on each individual pedigree

	Genome-wide maximum LOD score obtained from MERLIN-REGRESS by Pedigree^b^													
	
Gene^a^	1333	1340	1341	1345	1346	1347	1362	1408	1416	1418	1421	1423	1424	1454
*OR6A2*	0	0	0.29	0	0.11	0.56	0	0	0.18	0.21	**1.33**^c^	0	0.47	0
*NDUFB4*	0.07	0.36	0	0	0	0.37	0.08	0.04	0.06	0.33	**3.08**	0	0	**1.13**
*MAP3K6*	0.05	**1.56**	0.13	0	0	0	**1.03**	0	0.66	0.13	0	0	0.55	0.12
*RGS1*	0	0.14	**2.87**	0	0.69	0.29	0.21	0	0	0.86	0	0	0.05	0.14
*ARPC5L*	0	0.01	**3.81**	**1.14**	0.14	0.02	0	0.25	0.19	**1.11**	0	0	0	0
*CHI3L2*	**1.15**	0.49	**2.78**	**4.52**	0	**2.21**	0.03	0.27	**2.06**	**1.95**	0.05	0.30	0.79	0
*ITGB1BP1*	**4.75**	0.02	0	0.32	**2.59**	0	0.10	0	0	0.08	0	**2.60**	0.46	0.22
*HLA-DPB1*	0.08	0.13	0.07	0	0.77	**1.15**	**1.03**	0.30	**1.99**	0	0.69	0	0.40	**1.51**
*HLA-DQB1*	0	0	0.11	**1.18**	0.22	0	0.11	0.18	0.33	0	0.60	0.79	0.22	0.36
*ZP3*	0.29	0.8	0.01	0.21	0	0.39	0.14	0.78	**4.72**	0	0	**1.50**	**3.53**	**1.82**

^a ^Genes were selected as in Table 1.

^b ^The same SNP that mapped to the genome-wide maximum LOD score for each gene expression with all 14 pedigrees was used in linkage analysis on each individual pedigree

^c ^LOD > 1 is shown in bold font.

**Figure 3:**
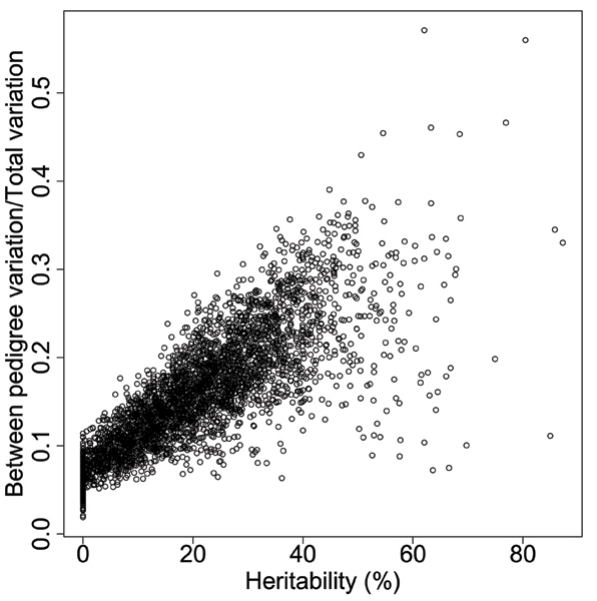
Comparison of the estimated heritability and the ratios of between pedigree variation versus total variation for 3554 genes (correlation = 0.81).

We also separated out 25 gene expressions with significant linkage signals but that have *h*^2 ^> 50%, and further studied the heritability difference between the two groups. We calculated the ratio of between-pedigree variation to the total variation, defined as the sum of between-pedigree variation and within-pedigree variation, and compared it to the heritability estimation (Fig. 3). A mean ratio of 0.09 was found for the group with *h*^2 ^< 10% (89 gene expressions), and a mean ratio of 0.30 for the group with *h*^2 ^> 50% (25 gene expressions). For all 3554 gene expressions, the mean ratio is 0.09 for the 880 gene expressions with *h*^2 ^< 10%, and 0.25 for the 108 gene expressions with *h*^2 ^> 50%. The heritability difference between these two groups suggests that the within-pedigree variation (e.g., environmental variance), may affect the heritability estimation more than the between-pedigree variation [12]. Therefore, heritability estimates without appropriate considerations of potential environmental contributors may lead to biased estimates of genetic contribution to a complex trait.

## Discussion

In this study, we have investigated the relationship between the estimated heritability of gene expression traits and the corresponding linkage signals using the CEPH population data provided by GAW15 Problem 1. This was motivated by a recent study using heritability as a filter for traits selection before conducting linkage analysis in a rat cross [7]. The rationale for this filtering is that the estimated heritability may be a good indicator of the statistical power to detect significant linkage regions for complex traits. The highly heritable traits may have more genetic effect contributing to their total phenotypic variance, hence significant linkage regions tend to be detected more easily. However, we found 89 expression traits with significant linkage signals that actually have small estimated heritability (*h*^2 ^< 10%). Among the 89 expression traits, 23 of them have estimated heritability of 0. Our study suggests that significant genome regions can be identified even for genes with low heritability estimates, indicating that heritability may not be a reliable predictor for linkage mapping results. A significant portion of eQTLs may be filtered out by a relatively low heritability cut-off, especially for *trans*-acting eQTLs (49%). Based on our analysis, we also found that in general, MERLIN-REGRESS gave a higher genome-wide maximum LOD score compared to the MERLIN variance-component method (Table 1), although a positive correlation (0.60) exists between the results from the two methods. However, for the subset of genes chosen in Tables 1 and 2, we did not observe any obvious potential influential factors, e.g., outliers, that cause the difference in results.

The results from linkage analysis on individual pedigrees indicate that a significant LOD score may result from a few individual pedigrees with strong linkage signals on gene expression traits with an overall low trait heritability estimation, i.e., the proportion of between pedigree variation to the total variation is small, while the linkage analysis is not affected by between pedigree variation.

## Conclusion

Highly heritable genes have a greater proportion of phenotypic variation explained by genetic effects and tend to have genomic regions showing significant linkage scores. However, some genes with low heritability also show high linkage scores, indicating that heritability is not a consistent predictor of eQTL mapping. Caution should be taken if we use inferred heritability as a filter of genes to conduct genome-wide linkage analysis.

## Competing interests

The author(s) declare that they have no competing interests.
